# Utilization and reproductive performance of gilts in large-scale pig farming system with different production levels in China: a descriptive study

**DOI:** 10.1186/s40813-021-00239-6

**Published:** 2021-12-13

**Authors:** Ran Guan, Wenchao Gao, Peng Li, Xuwei Qiao, Jing Ren, Jian Song, Xiaowen Li

**Affiliations:** 1 Shandong New Hope Liuhe Agriculture and Animal Husbandry Technology Co., Ltd, 6596 Dongfanghong East Road Yuanqiao Town, Dezhou, 253000 Shandong People’s Republic of China; 2 Sichuan New hope Animal Husbandry Technology Co., Ltd., of 4th Floor Building 1 No. 7, Hangkong Road Wuhou District, Chengdu, 610100 Sichuan People’s Republic of China; 3 Shandong Swine Herd Health Big Data and Intelligent Monitoring Engineering Laboratory, Dezhou University, Dezhou, 253000 Shandong People’s Republic of China

**Keywords:** Gilts, Reproductive performance, Utilization, Litter production

## Abstract

**Background:**

This study was to investigate the utilization and reproductive performance of gilts in large-scale pig farms. Data of this descriptive study included 169,013 gilts of 1540 gilts’ batches on 105 large-scale pig farms from April 2020 to March 2021. According to the upper and lower 25th percentiles of piglets weaned per sow per year (PSY) during the research stage, pig farms were divided into three productivity groups: high-performing (HP), intermediate-performing (IP) and low-performing (LP) farms. On the basis of breeds, LP (LP-Total) farms was further divided into LP-breeding pig (LP-BP) and LP-commercial pig (LP-CP) groups. Average utilization, estrus and first mating data was collected from a total of 1540 gilts’ batches. The age-related factors (introduction age, age at first estrus and age at first mating) and litter production (total number of piglets, number of piglets born alive and number of weaned piglets, as well as their proportion distribution) among HP and LP groups were compared. The litter production in different age groups were also analyzed.

**Results:**

The introduction age, mortality and culling rate of HP farms were lower compared with LP farms. Total number of piglets per litter, number of piglets born alive per litter and number of weaned piglets per litter in HP farms were significantly more than those of LP groups, respectively. The proportion distribution peaks of litter production in HP farms were shifted about two more than those in LP groups, respectively; and the proportion of low litter production (eight per litter or less) was lower than that in LP groups. The results of different age groups showed that total number of piglets per litter and number of piglets born alive per litter in 220–279 d were the most, while that of 370 d was the least.

**Conclusions:**

The overall utilization and reproductive performance of gilts in HP farms was better than those of LP farms. The difference in utilization was reflected in introduction source, culling rate and mortality. While the age at first estrus and first mating, breeds and litter production were the main differences for reproductive performance.

## Background

Gilts are the basis for maintaining fertility in large-scale pig farms, which is the largest category in the breeding herd, accounting for 18–20% [1]. When sows are culled from the pig herd due to high parities or low reproductive performance, gilts must be introduced to ensure the reasonable parity structure of the sows in the pig farm and the stability of production objectives. Well-raised gilts are expected to have a good mating rate, farrowing rate and litter production, even the lifetime performance and longevity [2–4].

Piglets weaned per sow per year (PSY) can be used as a benchmark to evaluate productivity and reproductive performance of sows, which varies in different countries [5]. Denmark has an average PSY about 30.9 [6], while in North America is 25.3 [7]. Incredibly, China, as the world’s largest consumer and producer of pork, has an average PSY of only about 20 [8, 9]. The productivity of pig farms in various country can also be divided into high and low levels. A study of high-performing (HP) farms in the United States showed that compared with ordinary farms, their farrowing rate was 9.0% higher, with 0.6 more piglets born alive per litter [10]. The high productivity of HP farms is mainly due to better development of gilts, better breeding management, more advanced productive technology and better piglets care during lactation [11]. Obviously, there are differences between pig farms of different production levels, but no research have been found on HP and low performance (LP) of Chinese pig farms.

Since the gilts are still in the growth stage, their physical development and reproductive performance are different from that of the sows [7, 12, 13]. To the best of our knowledge, the utilization and reproductive performance of gilts have not been entirely evaluated. Therefore, this study classified the whole surveyed pig farms into three productivity groups by PSY for a period of time (one year, from April 2020 to March 2021), and analyzed the production and reproductive performance of gilts’ batches and individual gilts, in order to provide a database for the production and management managers, so as to formulate more tailored policies.

## Methods

### Farm description

All pig farms studied (n = 105) were from 1274 pig farms of the same domestic large-scale breeding company in China that fulfilled the following inclusion criteria, which were (1) being continuous and stable production for more than half a year (no major business strategy adjustment or extensive disease epidemic (especially African swine fever)), (2) having a population of 1,000 or more productive sows, (3) using the internal data management system of the company. All of these farms applied automatic feeding system (The feed was transferred from galvanized sheet silo to stainless steel feeders (gilts) or DL6 feed doser (sows) through auger feed line controlled by feed line controller.) and mechanical ventilation system (climate controller for controlling fans of different sizes). At different growth stages, pigs were fed with the corresponding formula of standardized feed (According to the reference feeding amount, gilts and sows were fed the corresponding 12 kinds of feeds in the stages of nursery, growth, fattening, pregnancy and lactation) provided by the company’s internal feed factory. All farms used artificial insemination to mate gilts and sows, and 2-3 inseminations was carried out in each estrus cycle. The average stock of sows was 2660 ± 69.4, while the gilts’ stock was 324 ± 24.9. The average PSY was 19.9 ± 0.4.

### Categories and definitions

According to the upper and lower 25th percentiles of PSY (PSY = Number of weaned piglets/Days during the research stage * 365.25/Average number of sows) ranking by the internal data management system, pig farms were divided into three productivity groups: HP farms (PSY > 23.5), intermediate-performing (IP) farms (PSY 16.1-23.5), and LP farms (PSY < 16.1). LP farms were further divided into three groups by breeds: LP-Total (including pure, two-way crossbred and three-way crossbred), LP-breeding pig (LP-BP) groups (including pure and two-way crossbred) and LP-commerical pig (LP-CP) groups (only three-way crossbred). As there were no commercial pigs in HP farms, HP farms were not further classified.

Utilization of gilts was defined as the successful conception and entering the breeding cycle since the introduction. The non-productive days (NPDs) referred to other days except the production days, including mating to pregnancy loss, pregnancy loss to return-service, pregnancy loss to present/departure, weaning-mating, weaning to present/departure. Research stage was defined as the stage from April 2020 to March 2021. Other definitions were shown in Tables 1, 2 and 3.

**Table 1:** Average utilization of 169,013 gilts in 1540 gilts’ batches

		High-performing pig farms (n = 26)	Intermediate-performing pig farms (n = 53)	Low-performing pig farms (n = 26)
		Mean ± SEM	Mean ± SEM	Mean ± SEM
Number	Gilts’ batches	235	948	357
Source	Self-breeding^1^	73.0% ± 8.2%^ab^	80.8% ± 4.3%^a^	54.6% ± 6.7%^b^
	Internal introduction^2^	23.8% ± ± 7.8%	13.2% ± 3.3%	27.4% ± 6.2%
	External introduction^3^	3.2% ± 1.9%^a^	6.0% ± 2.4%^a^	18.0% ± 6.2%^b^
Introduction	Average introduction number of gilts	121 ± 6.94	83 ± 5.89	174 ± 16.21
	Average introduction age	202 ± 3.76^a^	237 ± 1.73^b^	224 ± 2.56^c^
Mortality	Mortality of gilts^4^	1.8% ± 0.2%^a^	1.7% ± 0.2%^a^	6.6% ± 1.0%^b^
	Total mortality^5^	5.6% ± 0.5%^a^	15.4% ± 0.9%^b^	19.1% ± 1.4%^c^
Culling	Culling rate of gilts^6^	9.4% ± 1.4%^a^	10.5% ± 0.8%^a^	14.9% ± 1.4%^b^
	Total culling rate^7^	25.9% ± 2.0%^a^	32.8% ± 1.1%^b^	40.4% ± 1.8%^c^

^1^Self-breeding: Gilts were bred and fed by pig farms themselves

^2^Internal introduction: Gilts were provided by other pig farms of the internal company

^3^External introduction: Gilts were provided by the pig farms of the external company

^4^Mortality of gilts = Deaths from introduction to pre-mating/Introduction number of gilts

^5^Total mortality: Mortality during the research stage, regardless of the production phase (pre-mating, mating, conception, farrowing or feeding) of gilts. Total mortality = Deaths during the research stage/Introduction number of gilts

^6^Culling rate of gilts = Number of culling gilts from introduction to pre-mating/Introduction number of gilts. The reasons for culling mainly included abnormal estrus, disease or physiological defects

^7^Total culling rate: Culling rate during the research stage, regardless of the production phase (pre-mating, mating, conception, farrowing or feeding) of gilts. Total culling rate = Number of culling gilts during the research stage/Introduction number of gilts

^a,b,c^Bars with different letters differ significantly (*P* < 0.05)

**Table 2:** Average estrus information of 100,811 estrus out of 112,157 gilts in 1540 gilts’ batches

	High-performing pig farms (n = 26)	Intermediate-performing pig farms (n = 53)	Low-performing pig farms (n = 26)
	Mean ± SEM	Mean ± SEM	Mean ± SEM
Number of gilts’ batches	235	948	357
Total number of estrus^1^	21,198	44,917	34,696
Proportion of first estrus^2^	62.4%^a^	92.7%^b^	91.7%^b^
Proportion of second estrus^3^	21.0%^a^	5.1%^b^	4.7%^b^
Proportion of third or more estrus^4^	16.6%^a^	2.2%^b^	4.6%^b^
Total estrus rate^5^	77.2% ± 2.2%^a^	78.1% ± 1.2%^a^	66.3% ± 2.1%^b^
Average times of estrus^6^	1.2 ± 0.05^a^	0.9 ± 0.01^b^	0.9 ± 0.03^b^
Average age of first estrus^7^	209 ± 5.79^a^	224 ± 3.18^b^	213 ± 5.32^b^

^1^Total number of estrus: The total number of gilts with estrus from introduction to pre-mating

^2^Proportion of first estrus: Average proportion of gilts with once estrus in all estrus gilts of each gilts’ batch

^3^Proportion of second estrus: Average proportion of gilts with twice estrus in all estrus gilts of each gilts’ batch

^4^Proportion of third or more estrus: Average proportion of gilts with three times or more estrus in all estrus gilts of each gilts’ batch

^5^Total estrus rate = Number of gilts in estrus/Total number of estrus

^6^Average times of estrus: Average estrus times of gilts before mating in each gilts’ batch

^7^Average age of first estrus: Average age of first estrus age before mating in each gilts’ batch

^a,b^ Bars with different letters differ significantly (*P* < 0.05)

**Table 3:** Average first mating information of 97,998 mating out of 112,157 gilts in 1540 gilts’ batches

	High-performing pig farms (n = 26)	Intermediate-performing pig farms (n = 53)	Low-performing pig farms (n = 26)
	Mean ± SEM	Mean ± SEM	Mean ± SEM
Number of gilts' batches	235	948	357
Total number of mating	19,819	43,867	34,312
Mating rate at first estrus^1^	54.9% ± 2.6%^a^	74.6% ± 1.2%^b^	63.9% ± 2.1%^c^
Mating rate at second or more estrus^2^	18.4% ± 1.9%^a^	2.6% ± 0.4%^b^	1.9% ± 0.5%^b^
Mating rate under 135 kg^3^	12.8% ± 1.7%^a^	20.0% ± 1.2%^b^	7.3% ± 1.2%^ac^
Mating rate between 135 and 145 kg^4^	47.9% ± 2.5%	50.6% ± 1.5%	50.5% ± 2.2%
Mating rate above 145 kg^5^	12.6% ± 1.6%^a^	6.6% ± 0.7%^b^	7.9% ± 1.3%^b^
Mating rate under 210 d^6^	7.2% ± 1.2%^a^	8.9% ± 0.8%^a^	13.6% ± 1.6%^b^
Mating rate between 210 and 240 d^7^	24.6% ± 1.9%	23.5% ± 1.2%	23.3% ± 2.0%
Mating rate above 240 d^8^	41.5% ± 2.3%^a^	44.8% ± 1.4%^a^	28.9% ± 1.9%^b^
Total average mating rate^9^	73.3% ± 2.2%^a^	77.2% ± 1.2%^a^	65.8% ± 2.1%^b^
Average times of estrus at first mating ^10^	2.4 ± 0.96^a^	0.9 ± 0.03^b^	1.2 ± 0.26^b^
Average age at first mating^11^	216 ± 5.99^a^	224 ± 3.21^b^	213 ± 5.33^ab^

^1^Mating rate at first estrus = Number of mating at first estrus/Total number of mating

^2^Mating rate at second or more estrus = Number of mating at second or more estrus/Total number of mating

^3^Mating rate under 135 kg = Number of mating at weight under 135 kg/Total number of mating

^4^Mating rate between 135 and 145 kg = Number of mating at weight between 135 and 145 kg/Total number of mating

^5^Mating rate above 145 kg = Number of mating at weight above 145 kg/Total number of mating

^6^Mating rate under 210 d = Number of mating at age under 210 days/Total number of mating

^7^Mating rate between 210 and 240 d = Number of mating at age between 210 and 240 d/Total number of mating

^8^Mating rate above 240 d = Number of mating at age above 240 d/Total number of mating

^9^Total average mating rate = Total number of mating/Total number of introduction

^10^Average times of estrus at first mating: Average times of estrus at first mating in each gilts’ batch

^11^Average age at first mating: Average age of gilts at first mating in each gilts’ batch

^a,b,c^Bars with different letters differ significantly (*P* < 0.05)

### Data collection, study design and exclusion criteria

The production data were uploaded to the internal data management system by each pig farm. All data belonged to the company. The researchers were authorized by the company’s production management department and digital technology department to obtain the production data in this study. This study was a descriptive study that analyzed a total of 169,013 gilts of 1,540 gilts’ batches in 105 large-scale (more than 1,000 sows) pig farms from April 2020 to March 2021. The data analysis of this study was divided into two levels: The utilization and reproductive data of gilts’ batches in different production levels was considered as batch level. The age related factors of gilts and litter production in different productive levels were measured as individual level. In order to observe the influence of breeds on age-related factors and litter production, the differences among HP, LP-Total, LP-BP and LP-CP farms were compared. The data of gilts’ batches was complete, without any removal. For gilts used to compare HP and LP farms, records were excluded if they met any of the following exclusion criteria: Total number of piglets per litter was zero (687 gilts); Number of piglets born alive per litter and number of weaned piglets per litter were more than 14 (If these two indexes were ≥15, it exceeded the number of 14 effective nipples of sows (the maximum), the surplus piglets were fostered to other sows with less litter size, resulting in the weaning number of the litter inconsistent with the actual size; or the data was incorrectly entered.) (546 gilts) and other incomplete data (865 gilts). Thus, 35,847 out of 37,045 gilts were used for individual level studies.

### Statistical analysis

Descriptive statistics were conducted using WPS Office Excel for Mac version 3.3.0 (Kingsoft Office Corporation, Beijing, China). The influence of breeds on age-related factors and litter production, and the litter production of gilts with different first mating days were analyzed using Graphpad Prism 7.0 (Graphpad Software, inc.San Diego, CA, USA). Tukey’s multiple comparisons test of Ordinary one-way ANOVA was used to study the average utilization, estrus and first mating data of gilts’ batches among HP, IP and LP farms. The same method was used for litter production of HP, LP-Total, LP-BP and LP-CP farms. Normal distribution test of litter production at different first mating days of gilts were performed by SPSS Statistics software 22.0.0 (IBM Corp. Released 2013. IBM SPSS Statistics for Mac, Version 22.0. Armonk, NY). *P* < 0.05 showed significant difference.

## Results

A total of 169,013 gilts of 1540 gilts’ batches of 105 large-scale pig farms were analyzed. The source, introduction, mortality and culling of gilts’ batches differed in HP, IP and LP farms (Table 1). The gilts of HP and IP farms were mainly from intra-company farms, most of them were self-sufficient, and less than 10% were from external sources. Nearly 1/5 of gilts in LP farms came from outside the company. The average introduction age of HP farms was the youngest among the three categories of farms. HP farms had the lowest mortality of gilts, total mortality, culling rate of gilts and total culling rate, although mortality of gilts and culling rate of gilts were not statistically different from IP farms.

The average estrus of 112,157 gilts of 1,540 gilts’ batches in different production levels showed statistical differences (Table 2). The proportion of first estrus of HP farms was significantly lower than that of IP and LP farms, but the proportion of more than second estrus was much higher. The total estrus rate of HP and IP farms was 77–78%,which was significantly higher than that of LP farms. Compared with IP and LP farms, HP farms had more average times of estrus and younger average age of first estrus.

The first mating information of 112,157 gilts of 1,540 gilts’ batches in different production levels were shown in Table 3. Compared with IP and LP farms, HP farms had lower mating rate at first estrous, but higher mating rate at second or more estrous. For mating weight, there were no differences between 135 and 145 kg, but mating rate under 135 kg in IP farms was significantly higher than that of HP and LP farms. By contrast, HP farms showed higher mating rate when the mating weight was above 145 kg. For mating age, compared with HP and LP farms, LP farms had higher mating rate under 210 d, lower above 240 d, and the total average mating rate was also lower. Compared with IP and LP farms, the average times of estrus at first mating in HP farms had a 1.2-1.5 times more. IP farms had the older average age at first mating.

Table 4 showed the difference among HP, LP-Total, LP-BP and LP-CP groups. LP-CP groups had the unique breed (three-way crossbred pigs) among all groups. LP-CP groups had older introduction age and older age at first estrus than other groups. Although age at first mating in HP farms and LP-Total groups differed significantly, there was no significant difference between LP-CP groups and HP farms.

**Table 4:** Average age-related and litter production of 35,847 gilts

	High-performing pig farms (n =26)	Low-performing pig farms-Total pigs (n =26)	Low-performing pig farms-Breeding pigs (n = 22)	Low-performing pig farms-Commercial pigs (n = 18)
	Mean ± SEM	Mean ± SEM	Mean ± SEM	Mean ± SEM
Number of gilts	11,833	24,014	10,952	13,062
Introduction age	201 ± 0.46^a^	210 ± 0.25^b^	208 ± 0.38^c^	211 ± 0.34^d^
Age at first estrus	242 ± 0.42^a^	252 ± 0.28^b^	249 ± 0.34^c^	254 ± 0.42^d^
Age at first mating	255 ± 0.38^a^	253 ± 0.28^b^	250 ± 0.35^c^	255 ± 0.42^a^
Total number of piglets born per litter	11.8 ± 0.02^a^	9.7 ± 0.02^b^	9.9 ± 0.03^c^	9.6 ± 0.02^d^
Number of piglets born alive per litter	11.0 ± 0.02^a^	8.7 ± 0.02^b^	8.9 ± 0.03^c^	8.6 ± 0.03^d^
Number of weaned piglets per litter	9.3 ± 0.04^a^	7.1 ± 0.03^b^	7.0 ± 0.03^c^	7.3 ± 0.03^d^

^a,b,c,d^ Bars with different letters differ significantly (*P* < 0.05)

**Fig. 1:**
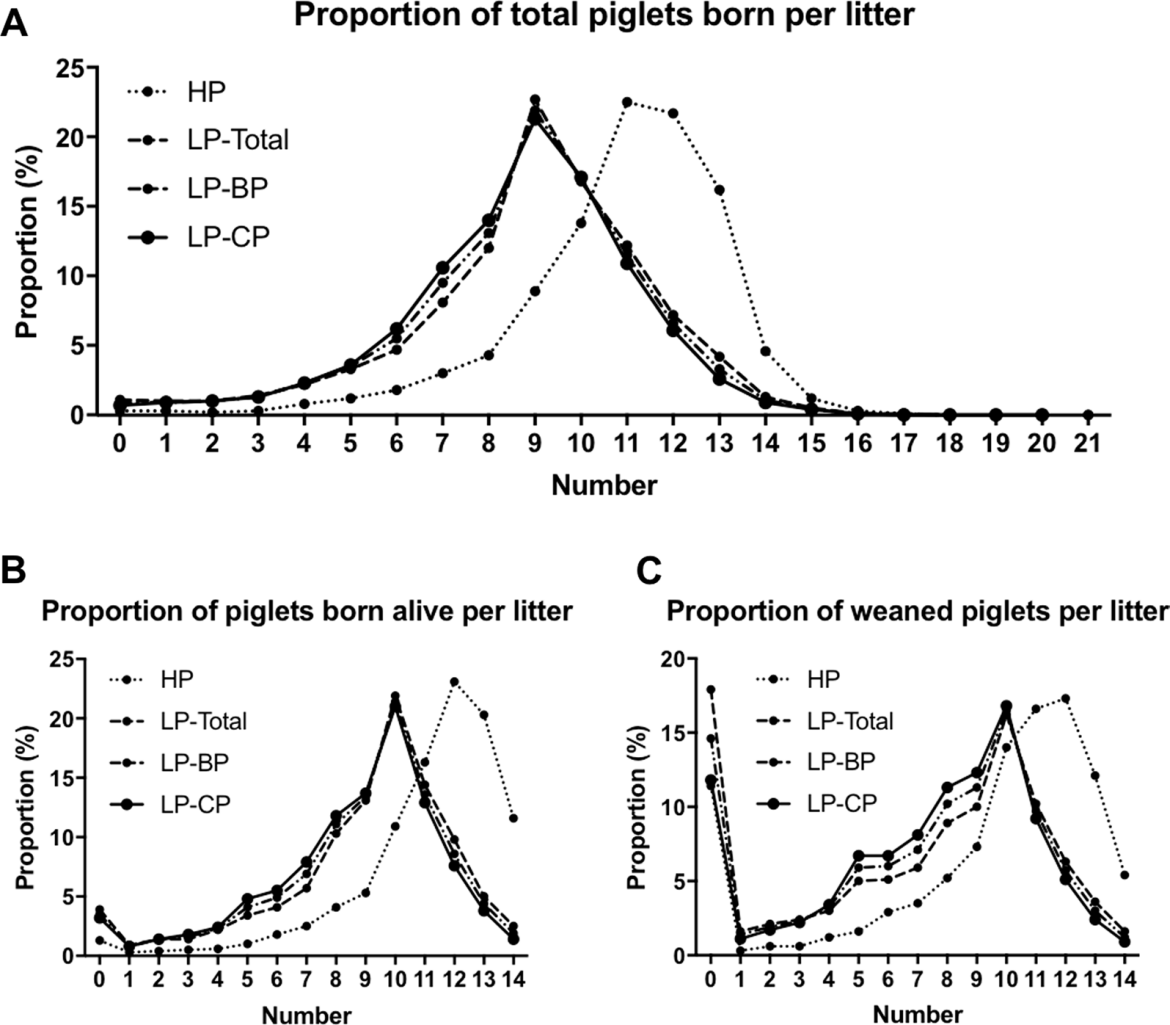
Litter proportion distribution of gilts at different production level. The proportion distribution peaks of litter production in HP farms were shifted about two more than those in LP groups, respectively; and the proportion of low litter production (eight per litter or less) was lower than that in LP groups. Values represent Mean ± SEM. (HP farms, n = 11,833; LP-Total groups, n = 24,014; LP-BP groups, n = 10,952; LP-CP groups, n = 13,062). ^a,b,c,d^Bars with different letters differ significantly (*P *< 0.05)

For litter production, total number of piglets per litter, number of piglet born alive per litter and number of weaned piglet per litter in HP farms were significantly higher than those of LP groups, respectively (Table 4). As shown in Fig. 1, the proportion distribution peaks of litter production in HP farms were shifted about two more than those in LP groups, respectively; and the proportion of low litter production (eight per litter or less) was lower than that in LP groups.

Table 5 showed the litter production of gilts with different first mating days. The data of total number of piglets born and number of piglets born alive at different first mating days of gilts were close to normal distribution. The Skewness were -0.619 and -0.794, respectively. The Kurtosis were -0.739 and -0.361, respectively. The total number of piglets born per litter and number of piglets born alive in 250-279 d and 340-369 d was the most, followed by 220-249 d. While the age above 370 d was the least under these two litter production parameters. The differences of number of weaned piglets were only seen in 190-219 d and 280-309 d, and no significant difference was found among other days.

**Table 5:** Litter production at different first mating days of 35,847 gilts

Age at first mating days	Number of gilts	Litter production		
		Total number of piglets born	Number of piglets born alive	Number of weaned piglets
		Mean ± SEM	Mean ± SEM	Mean ± SEM
160-189	898	9.9 ± 0.10^a^	9.1 ± 0.11^a^	7.9 ± 0.13^ac^
190-219	7,082	10.1 ± 0.03^a^	9.3 ± 0.04^ac^	7.9 ± 0.04^a^
220-249	10,575	10.5 ± 0.03^e^	9.6 ± 0.03^d^	7.8 ± 0.04^ac^
250-279	8,261	10.7 ± 0.09^d^	9.8 ± 0.03^e^	7.9 ± 0.05^ac^
280-309	5,522	10.3 ± 0.03^a^	9.2 ± 0.05^ac^	7.7 ± 0.06^bc^
310-339	2,259	10.3 ± 0.06^a^	9.3 ± 0.07^ac^	7.9 ± 0.08^ac^
340-369	651	10.8 ± 0.11^de^	9.6 ± 0.14^acde^	8.1 ± 0.17^ac^
370-399	347	9.3 ± 0.16^bc^	8.4 ± 0.18^b^	7.3 ± 0.21^ac^
≥400	252	9.5 ± 0.13^bc^	8.4 ± 0.18^b^	7.7 ± 0.21^ac^

^a,b,c,d,e^Bars with different letters differ significantly (*P* < 0.05)

## Discussion

This study clearly presented the multiple effects of pig farms with different production levels on the utilization and reproductive performance of gilts through statistical analysis of large amounts of data. The effects of utilization were mainly reflected in source, culling rate and mortality. While the age at first estrus, age at first mating, breeds and litter production were the main factors for reproductive performance. Among them, the negative effects of breeds (three-way crossbred) on conception rate, farrowing rate and litter production can not be ignored.

In our study, more than 90% of the gilts in HP pig farms and IP pig farms came from the internal company (self-breeding and internal introduction). For farrow-to-finish pig farms, self-breeding was a common way of introduction. It can not only cut the costs of gilt purchase, but also reduce the risk of new pathogens brought by external introduction [11, 14]. By comparison, 82% of the gilts in LP farms came from the internal company. This structure of source may increase the risk of introducing unknown diseases into LP farms.

Our study showed that the culling rate of gilts in LP farm was 14.9%, which was significantly higher than that in HP (9.4%) and IP pig farm (10.4%). The main reasons were undesirable limb configuration, repeated mating infertility and anestrus [13, 15, 16]. Tani et al. [17] suggested that when the age at first mating increased from 220 d to 300 d, the risk of culling of gilts due to reproductive failure increased by 2.1%. Because the average introduction age in LP farms was 223 d, the records of first induced estrus and subsequent estrus were missed. Therefore, it was easy to delay the best time of breeding, resulting in the cull of these gilts due to reproductive problems. In general, the annual renewal rate of gilts was 45–60%, which was a very important production cost of pig farms [18]. From an economic point of view, gilts must serve more than three parities in the breeding herd to reduce the substantial renewal costs [19]. On the premise of ensuring the parity structure of sows, the lower the renewal rate of gilts, the higher the profit of pig farm [20]. However, during the entire one-year research stage, the total culling rate of LP farms was as high as 40.4%. Together with the total mortality of 19.1% (Table 1), the loss of gilts was nearly 60%, indicating that the overall utilization of gilts in LP farms was very low.

Estrus was the basis for the reproductive performance of gilts. Gilts were bred in subsequent estrus cycles rather than their first cycle [21]. The breeding technical standard of gilts in our company was to introduce gilts (age at 130 d), then raised them in a pen with more than a dozen gilts, induced estrus at age of 160 d, recorded the first estrus, and transferred the estrus gilts to the single column. Afterwards, when the gilts aged at 210 d and weighed more than 135 kg, they can be bred for the first time. However, due to the shortage of sow source, the age of introduction/first mating in all groups was generally older, especially in LP-CP group. This not only caused the gilts to miss the optimal mating period (second or third estrus), increased the NPDs and feed consumption due to excessive feeding time, but also further reduced the estrus rate due to excessive weight gain [1, 22]. Gilts that missed the mating period will occupy a corresponding number of individual columns while waiting for the next estrus period. As a result, other breeding gilts that need to be transferred to individual columns can only continue to be raised and bred in pens. Once they fought with each other and caused severe acute stress, it continued to affect their reproductive performance, which not only reduced animal welfare, but also shortened the sow’s production life [23]. This study found that the total estrus rate and total mating rate of LP farms were lower than those of HP and IP farms, which may be one of the reasons for poor production performance.

An interesting finding of this study was that in all classified pig farms, only LP pig farms had three-way crossbred gilts, and the proportion was as high as 54%. It was reported that breeds had an important impact on the reproductive performance of sows [24]. In order to study the effect of breeds on the reproductive performance of gilts, LP farms was further divided into breeding pig groups (LP-BP) and commercial pig groups (LP-CP). The results showed that the average total number of piglets born per litter and average number of piglets born alive per litter of LP-CP groups were the least in all groups (Table 4). The proportion distribution peaks of litter production in LP groups were shifted about two less than those in HP farms, respectively; and the proportion of low litter production (eight per litter or less) was higher than that in HP farms (Fig. 1). This may be the characteristics of three-way crossbred pigs, that is, high growth performance and superior meat quality, but poor reproductive performance [25]. In March 2019, the price of pork in China continued to rise [26]. Coupled with the influence of African swine fever, there was an extreme shortage of breeding sows. In this case, a large number of three-way crossbred sows originally used as commercial pigs were used as breeding sows [27]. With the recovery of pig production, three-way crossbred sows, which were used as emergency supplements during special periods, were gradually culled.

We also found that there was a certain relationship between litter production and the age at first mating. When the age at first mating was 160–279 d, the average total number of piglets born per litter and average number of piglets born alive per litter increased with the increase of age, and decreased after 280 d, but there was a small peak at 340–369 d. This was inconsistent with the report of Saito [4], because he calculated the annualized lifetime number of pigs born alive. Nevertheless, the overall trend was similar.

This study had some limitations. The litter production was only statistically analyzed at the first parity. If the reproductive performance of all parities throughout the lifetime can be recorded, the impact of farm performance on the reproductive performance of gilts will be more accurately assessed. The current data represented many different management conditions and levels, ignoring the possible interaction between these factors. However, this study was worthwhile to describe the overall trend of production level of large-scale pig farms. The gap between HP farms and LP farms in terms of comprehensive utilization and reproductive performance of gilts was emphasized, which provided reference indicators for LP farms to be improved.

## Conclusions

There were differences in the overall utilization and reproductive performance of gilts in pig farms of different production levels. The production level of HP farms was significantly higher than that of LP farms. The differences in the overall utilization of gilts were mainly reflected in the introduction source, culling rate and mortality in the gilts stage and the research stage. The age at first estrus, the age at first mating, breed and litter production were the main factors that affected reproductive performance.

## Data Availability

Due to producer confidentiality, the dataset and farm information is not publicly available.
